# Effects of levosimendan on occurrence of cerebral vasospasm after aneurysmal subarachnoid hemorrhage: a case–control study

**DOI:** 10.1186/s13054-021-03824-x

**Published:** 2021-11-16

**Authors:** Antoine Trinh-Duc, Marc-Antoine Labeyrie, Anaïs Caillard, Wagih Ben Hassen, Alexandre Mebazaa, Benjamin Glenn Chousterman

**Affiliations:** 1 Department of Anesthesiology and Critical Care, DMU Parabol, FHU PROMICE, APHP.Nord, Lariboisière Hospital, 2 rue Ambroise Paré, 75010 Paris, France; 2 INSERM U942 MASCOT, Université de Paris, Paris, France; 3 Department of Interventional Neuroradiology, Hopital Lariboisière, Paris, France; 4 EA 7334 REMES, Université de Paris, Paris, France; 5 UMR 1266, Department of Neuroradiology, GHU Paris, Université de Paris, INSERM, Paris, France

**Keywords:** Subarachnoid hemorrhage, Cerebral vasospasm, Delayed cerebral ischemia, Levosimendan

## Dear Editor,

Delayed cerebral ischemia (DCI) is a common complication following aneurysmal subarachnoid hemorrhage (aSAH) contributing to poor prognosis [1]. Macro-vascular dysfunction related to cerebral vasospasm (CVS) remains the main pathophysiological hypothesis and therapeutic target of DCI [2]. No preventive treatment for CVS has yet proven to be effective [3].

Levosimendan, a non-adrenergic calcium-sensitizing inotrope, has been used to treat neurogenic stress-induced cardiomyopathy (NSIC) related to aSAH. Only few cases of patients treated with levosimendan for NSIC related to aSAH have been reported and have suggested that levosimendan could be associated with hemodynamic and neurological improvement [4]. The aim of this study was to retrospectively evaluate the efficacy of levosimendan as a therapy targeting occurrence of CVS in a cohort of patients admitted in ICU for aSAH.

We retrospectively reviewed the medical records of patients admitted to the Lariboisière Hospital Surgical Intensive Care Unit (ICU) (Paris, France) for an aSAH and treated with levosimendan during the first 48 h after ICU admission in order to treat neurogenic stress-induced cardiomyopathy (NSIC) secondary to aSAH (“Levo”) defined as an elevation of circulating troponin. We matched with a 1:2 ratio all Levo patients to patients that were not treated with levosimendan. Matching was performed on age, World Federation of Neurosurgical Societies (WFNS) grade, Fischer grade, need for external ventricular derivation (EVD) and severity assessed by Simplified Acute Physiology Score II (SAPS II). We excluded patients who died or had treatment withholding or withdrawal within the first four days following the bleeding. Levosimendan was administered intravenously as a continuous infusion over 24 h at a 0.1 μg/kg/min dose.

The primary endpoint of the study was the rate of occurrence of CVS defined as vessel narrowing higher than 50% requiring angioplasty and/or associated with DCI. DCI was defined according to Vergouwen et al. [5] Secondary outcomes included occurrence of DCI, 3-month modified Rankin scale (mRS) and variation of the cerebral arteries diameters between days 5–7 and day 1.

Between January 2018 and May 2020, a total of 652 patients were admitted for aSAH in our institution, and 18 Levo patients and 36 controls could be included in the study. The characteristics and outcomes of the 54 included patients are presented in Table 1.

**Table 1:** Characteristics, management and outcome for levosimendan cases and non-levosimendan controls

	Cases (“Levo”)^a^ (n = 18)	Controls^b^ (n = 36)	*P*-value	Odds-Ratio
Patients characteristics				
Age—median (IQR)—years	61 (50–70)	61,5 (52–68)	0.63	
Female—No (%)	15 (83.3)	30 (83.3)	1.00	
Arterial hypertension—No (%)	6 (33.3)	17 (47.2)	0.39	
Smoking—No (%)	10 (55.6)	10 (27.8)	0.07	
Diabetes—No (%)	0 (0)	1 (2.8)	1.00	
Stroke history—No (%)	2 (11.1)	4 (11.1)	1.00	
SAPS 2—median (IQR)	60 (43; 65)	49 (40; 57)	0.15	
SOFA—median (IQR)	8 (7; 11)	6 (2; 9)	0.03	
Characteristic of SAH				
WFNS grade—median (IQR)	5 (4; 5)	4 (4; 5)	0.43	
Fischer grade—median (IQR)	4 (4; 4)	4 (4; 4)	0.48	
Coiling—No (%)	16 (88.9)	32 (88.9)	1.00	
External ventricular drainage—No (%)	7 (38.9)	23 (63.9)	0.09	
Decompressive craniectomy—No (%)	2 (11.1)	3 (8.3)	1.00	
Cardiac impairment^c^				
Cardiac troponin peak—median (IQR)—ng/L	3999 (2210; 7382)	821 (40; 4532)	0.007	
BNP peak—median (IQR)—ng/L	1122 (762; 1718)	138 (65; 937)	0.001	
Outcome				
Cerebral vasospasm—No (%)	5 (27.8)	24 (66.7)	0.009	0.199 [0.0443; 0.766]
Delayed cerebral ischemia—No (%)	0 (0)	7 (19.4)	0.08	NA
MRS at 3 months ≤ 3—No (%)	11 (61.1)	18 (50)	0.57	1.558 [0.4331; 5.922]
Death at 3 months—No (%)	1 (5.6)	9 (25)	0.14	0.181 [0.004; 1.516]

Characteristics of patients are compared with Mann–Whitney U test for quantitative variables and Fisher’s exact test for categorical variables

BNP, B-type natriuretic peptide; MRS, modified Rankin score; NA, not available; SAPS2, Simplified Acute Physiology Score 2; SOFA, Sepsis-related Organ Failure Assessment; WFNS, World Federation of Neurosurgical Societies

^a^No missing data

^b^Missing data: arterial hypertension status for one patient, smoking status for one patient, diabetes status for one patient, stroke history for one patient, SOFA for six patients, type of aneurysmal securization for one patient, cardiac troponin peak for four patients, BNP peak for ten patients, use of norepinephrine for two patients

Considering the primary outcome of the study, the rate of CVS was lower in patients treated with levosimendan compared to the control group (5/18 (28%) vs 24/36 (67%), respectively, *p* = 0.009).

There was no brain infarction related to CVS in the Levo group, but the difference with the control did not reach statistical significance (0/18 (0%) vs 7/36 (19%) for the Levo vs control groups, respectively, *p* = 0.08).

There was no association between levosimendan administration and mRS at 3 months (OR 1.56 [0.43;5.92]; *p* = 0.56) neither for mortality at 3 months (OR 0.18 [0.00; 1.52]; *p* = 0.14).

**Fig. 1:**
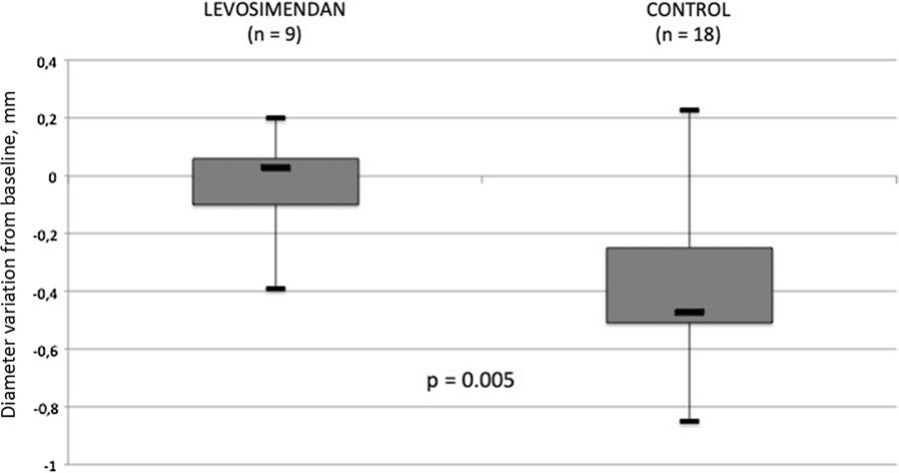
Mean vessels diameters variations between day 1 and days 5–7 in levosimendan treated vs control patients. Quantitative analysis of cerebral arteries diameters in patients who had vessel imaging (CT or angiography) at admission and at days 5–7 in nine levosimendan-treated patients and 18 controls. Vessels narrowing was more pronounced in controls compared to levosimendan-treated patients (*P* = 0.004)

Regarding evolution of vessels diameters, we observed a slight increase in the median cerebral arteries diameters between D0 and D5–7 (0.03 mm (IQR [− 0.01; 0.06])) in the Levo group, while cerebral arteries showed signs of vasospasm with a decreasing diameter in the control group (− 0.47 mm (IQR [− 0.51; − 0.24]) (*p* = 0.002; Fig. 1). Characteristics of the patients included in this analysis did not differ.

We found an association between the administration of levosimendan and a reduced incidence of CVS. Our study presents several limitations including its retrospective and non-randomized design that may induce several biases. Cardiac impairment was more frequent in the Levo group, since NSIC is associated with occurrence of CVS [6]; this argues in favor of a positive impact of levosimendan.

Our results suggest that levosimendan could represent a therapeutic candidate for early prevention of cerebral vasospasm secondary to aSAH.

## Data Availability

Data are available upon request to corresponding author.
